# Searching for prostate cancer by fully automated magnetic resonance imaging classification: deep learning versus non-deep learning

**DOI:** 10.1038/s41598-017-15720-y

**Published:** 2017-11-13

**Authors:** Xinggang Wang, Wei Yang, Jeffrey Weinreb, Juan Han, Qiubai Li, Xiangchuang Kong, Yongluan Yan, Zan Ke, Bo Luo, Tao Liu, Liang Wang

**Affiliations:** 1 Department of Radiology, Tongji Hospital, Huazhong University of Science and Technology, Jiefang Road 1095, 430030 Wuhan, China; 2 School of Electronics Information and Communications, Huazhong University of Science and Technology, Luoyu Road 1037, Wuhan, Hubei 430074 China; 3 Department of Nutrition and Food Hygiene, MOE Key Lab of Environment, Hubei Key Laboratory of Food Nutrition and Safety, Health, School of Public Health, Tongji Medical College, Huazhong University of Science and Technology, Hangkong Road 13, 430030 Wuhan, China; 4 Department of Radiology and Biomedical Imaging, Yale University School of Medicine, New Haven, 208042 Connecticut USA; 5 Department of Maternal and Child and Adolescent & Department of Epidemiology and Biostatistics, School of Public Health, Tongji Medical College, Huazhong University of Science and Technology, Hangkong Road 13, 430030 Wuhan, China; 6 Program in Cellular and Molecular Medicine, Boston Children’s Hospital, Boston, MA 02115 USA; 7 Department of Radiology, Union Hospital, Huazhong University of Science and Technology, Jiefang Road 1277, 430022 Wuhan, China; 8 School of mechanical science and engineering, Huazhong University of Science and Technology, Luoyu Road 1037, 430074 Wuhan, China; 9 Department of Radiology, Tongji Hospital, Tongji Medical College, Huazhong University of Science &Technology, Jie-Fang-Da-Dao 1095, Wuhan, 430030 P.R. China

## Abstract

Prostate cancer (PCa) is a major cause of death since ancient time documented in Egyptian Ptolemaic mummy imaging. PCa detection is critical to personalized medicine and varies considerably under an MRI scan. 172 patients with 2,602 morphologic images (axial 2D T2-weighted imaging) of the prostate were obtained. A deep learning with deep convolutional neural network (DCNN) and a non-deep learning with SIFT image feature and bag-of-word (BoW), a representative method for image recognition and analysis, were used to distinguish pathologically confirmed PCa patients from prostate benign conditions (BCs) patients with prostatitis or prostate benign hyperplasia (BPH). In fully automated detection of PCa patients, deep learning had a statistically higher area under the receiver operating characteristics curve (AUC) than non-deep learning (*P* = 0.0007 < 0.001). The AUCs were 0.84 (95% CI 0.78–0.89) for deep learning method and 0.70 (95% CI 0.63–0.77) for non-deep learning method, respectively. Our results suggest that deep learning with DCNN is superior to non-deep learning with SIFT image feature and BoW model for fully automated PCa patients differentiation from prostate BCs patients. Our deep learning method is extensible to image modalities such as MR imaging, CT and PET of other organs.

## Introduction

Prostate cancer (PCa) is a cause of death since ancient time documented in Egyptian Ptolemaic mummy imaging^1^. In the western countries, PCa is the most prevalent cancer and the third leading cause of cancer death in men^2^. In East Asian countries, the morbidity and mortality of PCa have increased dramatically, making it the fastest rising cancer among male malignancies. The accurate detection of PCa is an interesting but challenging task for physicians^3^. The distinction of PCa from prostate benign conditions (BC) including prostate benign hyperplasia (BPH) and prostatitis, is critical to personalized medicine^4^. Currently, MR Images of the prostate are manually evaluated by radiologists. However, the detection of PCa using MR images varies considerably. In a recent meta-analysis for PCa detection the specificity was 0.73 (95% confidence interval [CI] 0.60–0.83) with sensitivity of 0.89 (95% CI 0.86–0.92)^5^.

Machine learning (ML) is a branch of artificial intelligence (AI) that adopts various optimization, probabilistic, and statistical tools to learn from past examples and to then employ that prior training to classify new data, predict novel trends or identify new patterns^6–8^. Imaging recognition technology is one of the core technologies of ML^9^.

Traditional image recognition methods, such as Support Vector Machine (SVM), Artificial Neural Networks (ANNs), Bayesian Networks (BNs), Decision Trees (DTs), k-nearest neighbors (k-NN), and Adaboost^9–11^, use hand crafted image features, such as texture, shape, density of pixels, and off-shelf classifiers. We name these traditional imaging recognition methods as non-deep-learning methods in this paper. The non-deep-learning methods have been contributed to medicine, such as breast cancer, neurodegenerative diseases, psychiatric diseases, and others^7,8,11–14^. However, the main limitation of non-deep-learning methods is that these methods depend on the feature-extraction step and it is hard to find and extract suitable image features for a specific medical-image recognition problem. If the feature is not effective enough, the off-shelf classifier is not able to obtain satisfactory results.

The use of AI technologies in medical imaging has becoming popular, especially with the development of deep learning^15–27^. To deal with image data, deep convolutional neural network (DCNN) is used^28–33^. Deep learning methods enable to learn adaptive image features and perform image classification simultaneously. Considering significant achievement of deep learning on image recognition, it is potential to apply deep learning to classify MR images for automated disease assistant-diagnosis. To the best of our knowledge, this is not yet a study to compare deep-learning and non-deep-learning methods to medical imaging for the automatic classification of PCa or BCs patients with MR images.

The objective of the study is to search for PCa patients through MR imaging classification using deep learning versus non-deep learning. The choice of non-deep-learning algorithm is one challenge of this study. As a matter of fact, there have been many computer-aided diagnosis (CAD) algorithms for the prostate in MRI^7^. However, most of the CAD algorithms do not have source code and implementation details^7^. It is impossible to make comparison between studies without source code and implementation details. Thus, for a fair comparison we chose SIFT image feature with bag-of-word (BoW) model, a representative method in image recognition and analysis. In computer vision domain, the BoW was the representative method for CAD before the era of deep learning. Please refer to the image-net 2012 results^34^.

We compare a deep learning with DCNN algorithm and a non-deep learning with SIFT image feature and BoW model algorithm on building classifiers to distinguish PCa patients with prostate BCs patients using morphologic images (axial 2D T2-weighted imaging) of the prostate. We carry out a patient-based PCa and BCs classification, which only require patient-level labeling of the PCa or BCs patients and the corresponding images rather than segmenting the lesions on images in the learning algorithm. The gold standard used to assign the labels to the training images was the pathological diagnosis. The deep learning with DCNN method obtains significantly better than the non-deep learning with SIFT image feature and BoW model method. The deep learning with DCNN method may ultimately enable imaging interpretation to be easier and assist novices or general physicians to increase diagnostic accuracy, efficiency and consistency, and contribute to precision medicine.

## Results

**Figure 1:**
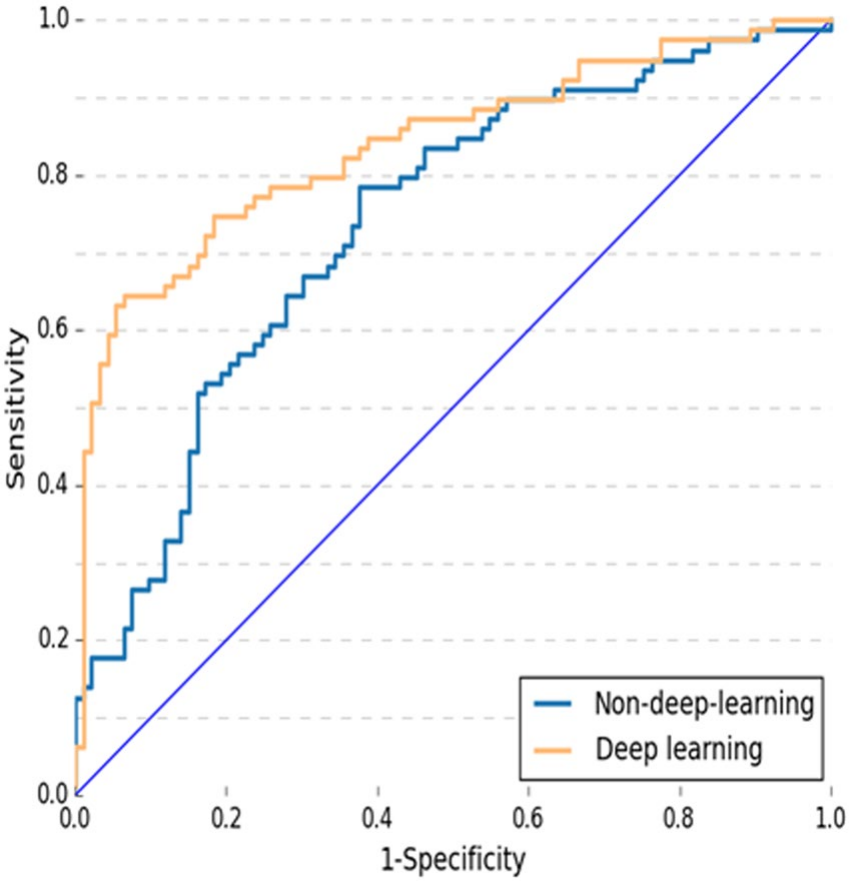
The ROC curves for PCa and prostate BCs patients differentiation of non-deep learning with SIFT image feature and BoW model versus deep learning with deep convolutional neural network (DCNN). Note: ROC curve: receiver operating characteristic curve. AUC: area under ROC. PCa = prostate cancer prostate BCs = prostate benign conditions BPH = benign prostatic hyperplasia.

To validate the effectiveness of the proposed deep learning method, comparison was made between deep learning with DCNN versus non-deep learning with SIFT image feature and BoW model in area under receiver operating characteristic curve (AUC), sensitivity, specificity, PPV and NPV. A *P* < 0.05 was considered statistically significant. Figure 1 and Table 1 provide overall PCa patients differentiation statistics of deep learning with DCNN versus non-deep learning with SIFT image feature and BoW model. The AUCs in deep learning with DCNN and non-deep learning with SIFT image feature and BoW model were 0.84 (95% CI 0.78–0.89) and 0.70 (95% CI 0.63–0.77) respectively. There was a significant difference between the AUCs in deep learning with DCNN and non-deep learning with SIFT image feature and BoW model (*P* = 0.0007 < 0.001). At the cut-off value above 0.5, sensitivity was 69.6%, specificity was 83.9%, positive prediction value (PPV) was 78.6%, and negative prediction value (NPV) was 76.5% for deep learning with DCNN. At the same cut-off value, sensitivity was 49.4%, specificity was 81.7%, PPV was 69.6%, and NPV was 65.5% for non-deep learning with SIFT image feature and BoW model.

**Table 1:** Overall PCa patient differentiation statistics of deep learning with DCNN versus non-deep learning with SIFT image feature and BoW model method.

Predictive test	AUC mean (95% CI)*	N (%) of PCa patient correctly classified			
		Sensitivity	Specificity	PPV	NPV
Deep learning	0.84 (0.78–0.89)	69.6% (55/79)	83.9% (78/93)	78.6% (55/70)	76.5% (78/102)
Non-deep-learning	0.70 (0.63–0.77)	49.4% (39/79)	81.7% (76/93)	69.6% (39/56)	65.5% (76/116)

Note: *P = 0.0007 < 0.001. Criterion: cut-off value >0.5. CI: confidence Interval. PPV: positive prediction value. NPV: negative prediction value. ROC curve: receiver operating characteristic curve. AUC: area under ROC. PCa = prostate cancer.

## Discussion

It is one of the first studies applying deep learning with DCNN as well as non-deep-learning to medical imaging. The novel study is a patient-based formulation for the automatic classification of PCa or prostate BCs patients with morphologic images (axial 2D T2-weighted imaging), in which a deep learning method and a non-deep learning method were compared. We develope a structure and workflow of deep learning that classifies PCa and prostate BCs patients based on the prostate morphologic images (axial 2D T2-weighted imaging), and compare deep learning classifiers with DCNN versus non-deep-learning with SIFT image feature and BoW model in the detection of PCa or prostate BCs patients.

Non-deep-learning methods^9–11^, such as SVM, ANNs, BNs, DTs, k-NN, and Adaboost, basically depend on a feature extraction step which usually describes images using texture, gradient and Gabor filters, etc. In contrast, it is a merit of our method that the proposed deep-learning method learns image features automatically within deep networks.

For a fair comparison, we chose SIFT features and BoW model as the representative of the so-called non-deep learning methods for image recognition. We extracted encoded the SIFT features using a pre-trained dictionary to generate a vector image representation. We applied a linear SVM classifier on the vector representation to obtain image classification. The SIFT features were extracted from the augmented images, which were generated using the same data augmentation method as we used in the deep learning method.

In our study, the training data and test data were better partitioned in terms of “subject”, i.e., patient. In this case, there were 172 subjects. In comparison to the recent ML papers on prostate studies, there were 147 subjects^9^ and 66 T2-weighted prostate images^35^, respectively. Even though this study was based on the ImageNet model, incorporating the features from the natural images, it should be noted that MR images are different from the nature images. It means that it may require a much larger sample size than 172. To make the subject sufficient for the study, our experiments were carried out according to 10-fold cross validation method published by Beheshti I^36^ and Ying J^37^. The method can ensure the robustness of our classifiers and the independence between the training data and test data. The pre-trained ImageNet model with augmented data was fine-tuned. As shown in the results, our deep learning classifier obtained robust performance. From the perspective of deep learning mechanism, the DCNN may work as a feature extractor, which provided a better feature for MR images. The final layer is a linear classifier which is equivalent to the classifiers in the non-deep learning method. As shown in the result, our method of deep learning from 172 subjects is feasible and interesting.

Deep learning that classifies image requires small-size images as the input of DCNN. A DCNN model that takes small-size image enables faster training and testing speed. We used the method published by Samala^33^, Rajkomar^32^ to resize each image with cubic interpolation. While preserving the original label during the training of deep learning classifier, we cropped each training image of 360 × 360 into multiple sub-images of 288 × 288. The method is popular in computer vision domain.

In this study, we showed that convolution features learnt from morphologic images (axial 2D T2-weighted imaging) of the prostate were used to classify PCa or prostate BCs patients. These convolution features were basically unrecognized by human inspection. However, the machine could efficiently and effectively recognize the convolution features and classified PCa or prostate BCs patients (Supplementary).

The deep learning system proposed in this paper relies on high-efficiency computational resources. Our experiments were carried out on a workstation with two Nvidia Titan X GPUs, an 8-cores Intel i7 CPU and 32GB memory. With these powerful computational resources, it took only 10 minutes to train a DCNN model for 1000 iterations and took less than 0.5 second to test a patient.

As described in the result section, the AUCs of deep learning with DCNN and non-deep learning with SIFT image feature and BoW model were 0.84 (95% CI 0.78–0.89) and 0.70 (95% CI 0.63–0.77) respectively. There was a significant difference between the AUCs in deep learning with DCNN and non-deep learning with SIFT image feature and BoW model (*P* = 0.0007 < 0.001). The sensitivity was 69.6%, specificity was 83.9%, positive prediction value (PPV) was 78.6%, the negative prediction value (NPV) was 76.5% for deep learning with DCNN. The sensitivity was 49.4%, specificity was 81.7%, PPV was 69.6%, NPV was 65.5% for non-deep learning with SIFT image feature and BoW model. Thus our deep learning with DCNN is able to produce better differentiation performance than non-deep learning with SIFT image feature and BoW model. Deep learning with DCNN has better capability in image recognition of prostate MRI than non-deep-learning with SIFT image feature and BoW model.

There are the limitations of the study. First, we use more than one image from each patient assuming independence among them, and classify PCa or prostate BCs patients with the images individually. Currently, in the research of computer vision and deep learning, understanding 3D data still lacks of effective methods. Analyzing 3d data in a frame by frame manner is the most popular way. In the future work, we will work on developing more effective deep learning methods by utilizing the dependence of the images from each patient for better PCa detection. Second, our study is a patient-based to compare deep-learning with non-deep-learning for the automatic classification of PCa or prostate BCs patients with morphologic images (axial 2D T2-weighted imaging). There was no manual work such as lesion-based label annotations or segmentation in our study. We believe that it is a merit of this paper. It has a clear advantage and makes sense in computer vision and deep learning. The advantage is that it does not require image label annotations or segmentations manually. Nevertheless, the patient-based study cannot provide information regarding tumor location on images. Third, our study merely used morphologic images (axial 2D T2-weighted imaging) of the prostate. The use of adding quantitative features from functional images including DWI and DCE-MRI remains to be investigated. Fourth, we merely focus on the comparison between deep learning with DCNN and non-deep-learning with SIFT image feature and BoW model. The comparison between deep learning and radiologist remains to be investigated. Nevertheless, the objective of our study is to determine which one of two methods, deep learning with DCNN versus non-deep learning with SIFT image feature and BoW model, is better in the detection of PCa. We found deep learning with DCNN is able to produce better differentiation performance than non-deep learning with SIFT image feature and BoW model in the detection of PCa patients.

In summary, we elucidated that prostate MR image machine learning classifiers in terms of morphological information can successfully search for PCa patients. This deep learning with DCNN method outperforms non-deep-learning with SIFT image feature and BoW model for the automatic classification of MR images of PCa or BCs patients. Our prostate MR images obtained from routine clinical practice, our machine learning classifiers could be efficiently implemented into current clinical practice. Accurate diagnosis provided by our deep learning with the DCNN method can contribute to precision medicine.

## Methods

### Subject

The research protocol for this retrospective study was approved by the institutional review board of Tongji Hospital, Tongji Medical College, Huazhong University of Science and Technology. All methods were performed in the principles of the Declaration of Helsinki. Written informed consent was obtained from the subject. 172 patients with 2,602 morphologic images (axial 2D T2-weighted imaging) of the prostate were obtained from Tongji Hospital, Tongji Medical College, Huazhong University of Science and Technology, with pathologically confirmed PCa and prostate BCs including BPH and prostatitis. The patient’s cohort included 93 prostate BCs and 79 PCa patients. Patient characteristics of the cohort are summarized in Table 2.

**Table 2:** Patient Characteristics.

Characteristics	Summary
PCa patients	N = 79
Age (years) (average, range)	67.9(50–88)
Number of MR imaging *	N = 1164
Prostate BCs patients	N = 93
Age (years) (average, range)	N = 66.5(47–91)
Number of MR imaging*	N = 1438
BPH	N = 75
BPH + prostatitis	N = 18

Note: PCa = prostate cancer. prostate BCs = prostate benign conditions. BPH = benign prostatic hyperplasia. *Morphologic images (axial 2D T2-weighted imaging). The study is a patient-based to to compare deep-learning with DCNN versus non-deep-learning with SIFT image feature and BoW model for the automatic classification of PCa or prostate BCs patients with morphologic images (axial 2D T2-weighted imaging).

### MR Image Acquisition

All MR images were performed by using a 3.0 Tesla (T) whole-body unit (MAGNETOM Skyra, Siemens Medical Solutions, Erlangen, Germany) followed by pathology reports and one year’s follow-up. The acquisition protocol included T2-weighted imaging (T2WI), T1-weighted imaging (T1WI), dynamic contrast-enhanced MRI (DCE-MRI) and diffusion-weighted imaging (DWI). The final cohort size was 172 patients with 2,602 morphologic images (axial 2D T2-weighted imaging) of the prostate used in our analysis (Table 2). The acquisition parameters for obtaining the 2dimensional transverse T2WI turbo spin-echo images were set as follows: repetition time (TR) 6750 ms, echo time (TE) 104 ms, echo train length 16, section thickness 3 mm, no intersection gap, field of view (FOV) 180 mm × 180 mm, image matrix 384 × 384. Most CNN networks require square images. In the paper, we resize the image to 360 × 360 before input the image into deep networks. The network we designed requires input image in this size. The common practice in deep learning helps to improve the speed of deep learning classifier.

The method published by Samala^33^, Rajkomar^32^ was applied to resize each image with cubic interpolation. The method could ensure accelerated our training and testing DCNN, while preserving the original label during the training of deep learning classifier. Each image of 360 × 360 was cropped into multiple sub-images of 288 × 288. Imaging processing and imaging analysis used all the images. For convenience, we set the images to have equal weight. The final classification result was obtained via a voting strategy formatted in Equation ${P}_{i}=\frac{1}{{M}_{i}}{\sum }_{j=1}^{{M}_{i}}{p}_{ij}$.

### Clinical variables and pathology reports

The clinical variables and pathology reports of each PCa patients and prostate BCs patients were acquired from our research database (Table 2).

### Cross-Validation

For the robustness of our classifiers, a 10-fold cross validation method published by Beheshti I^36^ and Ying J^37^ was applied to randomly partition the 172 patients into 10 equal sized groups to ensure the independence between the training data and test data, as described in Table 3.

**Table 3:** Training data, testing data of 10-fold cross validation on PCa and BCs patients differentiation experiments of deep learning with deep convolutional neural network (DCNN) versus non-deep-learning with SIFT image feature and BoW model.

No. of group		1	2	3	4	5	6	7	8	9	10
Training (No. of patients and No. of images)	All	155 (2342)	155 (2352)	154 (2310)	155 (2342)	155 (2318)	155 (2372)	155 (2371)	154 (2324)	155 (2350)	155 (2337)
	BC	84 (1295)	82 (1272)	84 (1283)	81 (1252)	86 (1314)	83 (1298)	88 (1370)	81 (1252)	82 (1276)	86 (1330)
	PCa	71 (1047)	73 (1080)	70 (1027)	74 (1090)	69 (1004)	72 (1074)	67 (1001)	73 (1072)	73 (1074)	69 (1007)
Testing (No. of patients and No. of images)	All	17 (260)	17 (250)	18 (292)	17 (260)	17 (284)	17 (230)	17 (231)	18 (278)	17 (252)	17 (265)
	BC	9 (143)	11 (166)	9 (155)	12 (186)	7 (124)	10 (140)	5 (68)	12 (186)	11 (162)	7 (108)
	PCa	8 (117)	6 (84)	9 (137)	5 (74)	10 (160)	7 (90)	12 (163)	6 (92)	6 (90)	10 (157)

Note: PCa = prostate cancer. prostate BCs = prostate benign conditions.

### Formulation of Image Classification using Deep Learning with DCNN

To describe the MR image classification using deep learning in the patient-based study, we gave the formulations in this section. At first, we denoted a patient as ${X}_{i}=\{{x}_{ij},j\in [1,\mathrm{..}.,{M}_{i}]\}$, where ${X}_{i},i\in [1,\mathrm{..}.,N]$ denoted the i-th patient, there were N patients in total, the i-th patient contains *M*_*i*_ images, and *x*_*ij*_ was the j-th image of the i-th patient. Then, each patient had a clinical label. Accordingly, each patient was denoted as *X*_*i*_ which means that we treated all images of a patient as a whole, each lesion of the patient will be either used for training or for testing in one fold of the experiments.

For the patient *X*_*i*_, its clinical label was denoted as *Y*_*i*_. We defined that if the i-th patient was PCa, *Y*_*i*_ = 1 and if the i-th patient was BCs, *Y*_*i*_ = 2.

Training a deep learning model required the label of each image, which meant we needed the label of *x*_*ij*_ denoted as *y*_*ij*_. To address the issue, we set the image label as its patient label, denoted as *y*_*ij*_ = *Y*_*i*_.

After obtaining the labels of all training images, we trained a deep CNN model using back-propagation in the Caffe deep learning toolbox. The learned deep CNN model was denoted as *f*.

In testing phase, we used the learned deep CNN model *f* to compute the classification probability *p*_*ij*_ of image *x*_*ij*_ denoted as *f*(*x*_*ij*_). *P*_*ij*_ was a vector denoted as ${p}_{ij}\in {R}^{K\times 1}$, where *K* was the number of classes. In this study, *K* = 2, *p*_*ij*_(1) was the probability *x*_*ij*_ belonged to prostate PCa, and *p*_*ij*_(2) was the probability *x*_*ij*_ belonged to BCs, and the sum of *p*_*ij*_ was 1 which was restricted in by the deep CNN structure.

After obtaining the classification result of each PCa or prostate BC patient with images, we could easily obtain the classification result of a patient by averaging its images classification results, denoted as ${P}_{i}=\frac{1}{{M}_{i}}{\sum }_{j=1}^{{M}_{i}}{p}_{ij}$. *P*_*i*_ was a *K* = 2 dimensional vector and its sum was equal to 1. Finally, we could get classification label of the patient *X*_*i*_ by checking which dimension had larger probability. If the first dimension had large value, it was classified as PCa patient; otherwise, it was classified as BC patient.

### Deep Learning Toolbox

In this study, a popular deep learning toolbox created by Berkeley Vision and a learning center, named Caffe, was utilized for implementing the automated PCa and BCs patient differentiation system (Supplementary). Python for data preparation, analysis and visualization was used. The hyper- parameters of our DCNN were set as follows: gamma 0.1, momentum 0.9, weight decay 0.1, and maximum training iteration 1000 (Supplementary).

### Training Deep Learning Classifier

**Figure 2:**
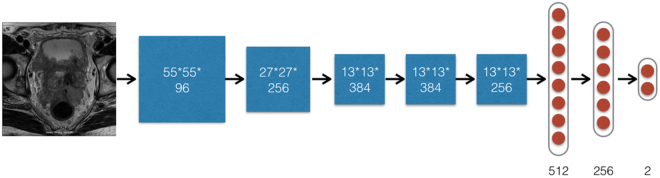
The structure of deep learning with deep convolutional neural network (DCNN) for the automatic classification of a PCa or BCs patient with morphologic images (axial 2D T2-weighted imaging). A 288 × 288 × 3 MR image was input. Five convolution layers and two inner product layers with sizes were shown in the figure. A max-pooling layer and non-linear ReLU layer following each convolution layer. A max-pooling layer downsize feature map gradually as demonstrated. Finally, an output layer specified PCa patient probability on input image. Note: PCa = prostate cancer. Prostate BCs = prostate benign conditions. BPH = benign prostatic hyperplasia.

The method published by Samala^33^, Rajkomar^32^ to resize each image with cubic interpolation was applied. A pre-trained ImageNet model was used for fine-tuning our model with augmented data. In data augmentation, each training image of 360 × 360 was cropped into multiple sub-images of 288 × 288 while preserving the original label during the training of deep learning classifier. The deep learning classifiers were used to conduct supervised machine learning. The inputs to the classification algorithms were the prostate morphologic images (axial 2D T2-weighted imaging). There were 5 convolutional layers and 2 inner product layers. After every convolutional layer, there was a max-pooling layer and a non-linear ReLU layer, respectively. Thus, different from the non-deep learning, which depends on the feature extraction step, DCNN automatically learnt from image features using the image labels given by the pathological diagnosis. The learned features were adaptive and more suitable for solving the problem without of human design. The outputs specified the predicted diagnosis groups. The hyper-parameters were set based on our experiences of tuning deep networks for image recognitions are given in Fig. 2.

### Formulation of Image Classification using Non-deep-learning Method

We used a non-deep-learning method named as bag-of-word (BoW) with SIFT image feature, a representative method for image recognition. SIFT image feature is popular for its scale and rotation invariance, it builds local image representation based on image gradient. For each image, a set of SIFT features were extracted and encoded using a pre-trained dictionary. The BoW model aggregated the encoded SIFT features into a vector representation for each image. Image classification was done with a linear SVM classifier on the vector representation. In addition, for a fairy comparison between deep learning and non-deep-learning, the SIFT features were extracted from the augmented images, which were generated using the same data augmentation method as we used in the deep learning method. Thus, for deep learning and non-deep-learning, the data augmentation method was the same. Then, we gave the formulation of BoW using SIFT feature as follows. We denoted the SIFT features extracted from MR image *x*_*ij*_ as *S*_*ij*_. *S*_*ij*_ were a set of feature vectors. Then we applied the Fisher vector coding^38^ to aggregate the local image descriptors into a vector representation of image *x*_*ij*_, denoted as ${F}_{ij}=FV({S}_{ij})$.

Based on the vector representations, we used SVM as the classifier of the studied non-deep-learning method, which was learned from training images and applied to classify testing images. For each training image, SVM classifier gave its probability of being PCa patient. We subsequently used the same equation for deep learning method (in Eq. (${P}_{i}=\frac{1}{{M}_{i}}{\sum }_{j=1}^{{M}_{i}}{p}_{ij}$) to calculate the probability of a PCa patient.

### Non-Deep-Learning Toolbox

To implement the proposed non-deep-learning method, the VLFeat toolbox was used to extract SIFT feature and Fisher vector coding and the LibLinear toolbox to do SVM training and testing. We used the same data augmentation method used in the deep learning method to process the training images. The hyper-parameters were given as follows: the number of clustering center of Fisher vector coding was set to 64. The bias term of linear SVM was set to be true.

### Training data, testing data of 10-fold cross validation on the PCa and prostate BCs patients differentiation experiments with deep learning versus non-deep-learning

For the robustness of our classifiers, we applied a 10-fold cross validation method published by Beheshti I^36^, Ying J^37^ to randomly partition the 172 patients into 10 equal sized groups. Specifically, as described in Table 3, the 10-fold cross-validation method ensured the independence between the training data and test data for deep learning with DCNN versus non-deep-learning with SIFT image feature and BoW model. Please refer to the source code in the Supplementary.

### Assessment

For diagnostic classification to differentiate PCa patients from prostate BCs patients based on deep learning with DCNN versus non-deep-learning with SIFT image feature and BoW model, ROC curves were performed and AUCs of every classifiers based on deep learning with DCNN versus non-deep-learning with SIFT image feature and BoW model were generated.

## Electronic supplementary material

Source code
