# Supplementary material for: Searching for prostate cancer by fully automated magnetic resonance imaging classification: deep learning versus non-deep learning
Source: Sci Rep. 2017 Nov 13;7:15415. doi: 10.1038/s41598-017-15720-y (PMC5684419; doi:10.1038/s41598-017-15720-y)
Supplement: Supplementary file 1 — Source code [file 41598_2017_15720_MOESM1_ESM.doc]

**Searching for prostate cancer by fully automated magnetic resonance imaging classification: deep learning versus non-deep learning**

Xinggang Wang Ph.D^1,2^, Wei Yang M.D^3^, Jeffrey Weinreb MD^4^, Juan Han MD,PhD^5^, Qiubai Li, MD^6^, Xiangchuang Kong M.M^7^, Yongluan Yan B.E^2^, Zan Ke M.M^1^, Bo Luo Ph.D^8^, Tao Liu Ph.D^8^, Liang Wang M.D, Ph.D^1*^

**Supplementary: Source code available at** [**http://mclab.eic.hust.edu.cn/~xwang/release/supp2%20source%20code%20and%20deep%20learning%20configuration%20file.zip**](http://mclab.eic.hust.edu.cn/~xwang/release/supp2 source code and deep learning configuration file.zip)

| Index | File name | Usage |
| --- | --- | --- |
| 1 | scripts/k_fold_split.py | Randomly splitting data into 10 folds |
| 2 | scripts/convert_images.sh | Converting images into the lmdb format |
| 3 | scripts/train.sh | Training using caffe |
| 4 | scripts/eval.py | Evaluate the learned caffe model and save results |
| 5 | scripts/read_result.py | Read results and calculate P-value |
| 6 | scripts/plot_error_bar.py | Code for plotting Figure in the paper |
| 7 | scripts/make_tab.py | Code for making Table in the paper |
| 8 | Models/* | Caffe configuration files |
